# *Clostridium botulinum* Strain Af84 Contains Three Neurotoxin Gene Clusters: *Bont/A2*, *bont/F4* and *bont/F5*

**DOI:** 10.1371/journal.pone.0061205

**Published:** 2013-04-12

**Authors:** Nir Dover, Jason R. Barash, Karen K. Hill, Karen W. Davenport, Hazuki Teshima, Gary Xie, Stephen S. Arnon

**Affiliations:** 1 Infant Botulism Treatment and Prevention Program, California Department of Public Health, Richmond, California, United States of America; 2 Bioscience Division, Los Alamos National Laboratory, Los Alamos, New Mexico, United States of America; Institute Pasteur, France

## Abstract

Sanger and shotgun sequencing of *Clostridium botulinum* strain Af84 type Af and its botulinum neurotoxin gene (*bont*) clusters identified the presence of three *bont* gene clusters rather than the expected two. The three toxin gene clusters consisted of *bont* subtypes A2, F4 and F5. The *bont/A2* and *bont/F4* gene clusters were located within the chromosome (the latter in a novel location), while the *bont/F5* toxin gene cluster was located within a large 246 kb plasmid. These findings are the first identification of a *C. botulinum* strain that contains three botulinum neurotoxin gene clusters.

## Introduction

Botulinum neurotoxin (BoNT), the most poisonous substance known, exists as seven toxin types A-G that are distinguished by the inability of polyclonal antibodies specific for one toxin type to neutralize any of the other six toxin types [1]. Variants or subtypes within BoNT/A, B, E and F have been designated by adding an Arabic number to the toxin type, e.g., A1, A2, etc. [2]. Some *C. botulinum* strains produce two botulinum toxin types, e.g., Ab, Ba, Af and Bf, with the larger amount of toxin produced indicated by a capital letter [3]–[11]. Additionally, some type A strains contain a complete but inactive type B neurotoxin gene designated A(B), while others contain a partial type B toxin gene designated A(B′) [6], [12].

The botulinum toxin (*bont*) gene is associated with accessory genes or predicted open reading frames (*orf*s) that collectively are referred to as the *bont* gene cluster. There are two different clusters: the hemagglutinin (*ha*) toxin gene cluster is found in strains that produce toxin types A1, A5, B, C, D, and G, while the *orfX* toxin gene cluster is found in strains that produce toxin types A1 to A4, E, and F [13]–[15]. The genomic location of the *bont* gene cluster may be either within the chromosome, a phage or a plasmid [15]–[17]. In bivalent strains with two *bont* gene clusters, the *bont* gene clusters have both been located either within the chromosome or both located within a plasmid [15], [18]. The locations of the *bont* gene clusters appear to be conserved; for instance, in *C. botulinum* proteolytic (Group I) strains the *bont* gene cluster is found at specific locations in the chromosome or within a plasmid [18].

Recognition of the unique clinical features of type F (*C. baratii* subtype F7) infant botulism (very young age at onset, very rapid onset, very severe paralysis, very rapid recovery) [19]–[25] prompted our interest in the characteristics of the multiple variants (subtypes) of type F toxin, the regulation of its expression, and the variation of its toxin gene clusters [5], [13]. The initiation in 2006 of collaborative genomic studies of *C. botulinum* and neurotoxigenic *C. baratii* by the California Department of Public Health (CDPH) and Los Alamos National Laboratory (LANL) enabled investigation of various botulinum toxin type F-producing strains in the CDPH collection, one of which was proteolytic (Group I) *C. botulinum* type Af strain Af84 (Argentina) that was a personal gift from its co-discoverer, D. Giménez, during his visit to CDPH in August 1979 [26]. The *bont/A2* and *bont/F4* genes have already been identified in strain Af84 [2], [3] (GenBank accession numbers FJ968749 and FJ968748, respectively). Recent characterization of the *bont* gene diversity of six additional bivalent Af strains from Argentina found that three contained the *bont/F4* gene and three contained the *bont/F5* gene. All six Af strains also contained the *bont/A2* gene [3].

Here we report the analyses of Sanger and shotgun sequencing of the original *C. botulinum* strain Af84 type Af provided to us in 1979. Unexpectedly, we found that the strain contains not just two, but rather three, botulinum neurotoxin genes, *bont/*A2, *bont/F4* and *bont*/*F5*. Each of the three toxin genes of strain Af84 was within a complete *orfX* toxin gene cluster; none were part of a *ha* toxin gene cluster. The *bont/A2* and *bont/F4* genes were located in the chromosome (with the *bont/F4* cluster in a novel location), while *bont/F5* was located in a large 246 kb plasmid.

## Materials and Methods

### Bacterial strains and culture conditions

The original *C. botulinum* strain Af84, isolated in 1966 from Argentinian soil by Giménez and Ciccarelli [8], [26], was the personal gift of D. Giménez to CDPH in 1979. The strain remained lyophilized in its original glass ampule until July 2009, when the ampule was aseptically opened and rehydrated with 2 ml of Trypticase-Peptone-Glucose-Yeast extract broth (TPGY). The suspension was incubated in TPGY broth anaerobically for 48 hours at 35°C. The broth was sub-cultured onto 4% Egg Yolk Agar plates and incubated for 48 hours at 35°C. A single colony, widely separated from the others on the plate, was then incubated in Chopped-Meat-Glucose-Starch (CMGS) broth for 48 hours at 35°C. Culture stock and spore suspension were prepared and stored at −75°C.

### Mouse protection assay

A tube containing pre-reduced CMGS broth was inoculated with 50 µl of a spore suspension of *C. botulinum* strain Af84 and was incubated at 35°C for 72 hours. The broth culture was centrifuged to remove the bacterial cells and meat particles and the resulting supernatant was filter-sterilized through a 0.45 µm PES filter membrane to create the final sterile culture filtrate, which was maintained at 4°C.

The culture filtrate preparation was evaluated with the standard mouse protection assay using the CDC antitoxins [27], [28] to verify the presence of BoNT/A and BoNT/F and to determine the relative ratio of their toxicities. Either 0.2 ml of antitoxin A or 0.2 ml of antitoxin F was mixed with 1.0 ml of diluted culture filtrate. Pairs of mice were inoculated (intraperitoneal route) with 0.6 ml per mouse of either the antitoxin A-treated or antitoxin F-treated diluted culture filtrate of strain Af84. Additionally, 0.2 ml antitoxin A and 0.2 ml antitoxin F were both combined with 1.0 ml of diluted culture filtrate and pairs of mice were inoculated with this mixture. Control mice were inoculated with 0.5 ml of culture filtrate with no antitoxin. Toxin activity was recorded as mouse death or survival up to 96 hours after inoculation.

The BoNT/A: BoNT/F ratio was determined by comparing the activity of the toxins contained in two parallel sets of 10-fold serial dilutions of culture filtrate; one set was absorbed with monovalent antitoxin A (in order to measure the amounts of type F toxin activity), while the second set was left unabsorbed (in order to measure the combined amounts of type A and type F toxins activities). Each dilution was made using gelatin phosphate diluent and was tested in groups of 4 Swiss-Webster mice (17–22 g).

All work was conducted under CDPH AUP# 12-02 and adhered to all CDPH Institutional Animal Care and Use Committee guidelines.

### DNA extraction

Genomic DNA for PCR and Sanger sequencing was extracted from cell pellets using the MagNA Pure Compact Nucleic Acid Isolation Kit I and the Bacteria Purification Protocol (Roche Applied Science, Indianapolis, IN) according to the manufacturer's instructions. Genomic DNA for next-generation sequencing was extracted using the Qiagen DNeasy Blood and Tissue Kit (Qiagen Inc, Valencia, CA) according to the protocol for Gram-positive bacteria. Genomic DNA for screening of multiple bacterial colonies of *C. botulinum* strain Af84 was extracted from 10 well-isolated individual bacterial colonies grown on 4% Egg Yolk Agar plates. The DNA was extracted with PrepMan Ultra Sample Preparation Reagent (Life Technologies, Carlsbad, CA) according to the manufacturer's instructions.

### DNA sequencing and analysis

*C. botulinum* strain Af84 was sequenced by combining Sanger sequencing of PCR products and next generation platforms that included the 454 Titanium [29] and Illumina [30] technologies. The data were assembled using Velvet version 0.7.63 [31] and Newbler version 2.3 [29]. Gaps between contigs were closed by editing in Consed [32]–[34] and by PCR. The final assembly consisted of a chromosome of 42 contigs (3,840 kb) and one plasmid (246 kb) that totaled 4,086,083 bp. This whole genome shotgun project has been deposited at DDBJ/EMBL/GenBank under accession number AOSX01000000.

The genome project was designated CLQ (and the plasmid pCLQ) for sequencing and annotation. The genome was annotated using the RAST server [35] and the Prokaryotic Genomes Automatic Annotation Pipeline (PGAAP) server at NCBI (http://www.ncbi.nlm.nih.gov/genomes/static/Pipeline.html). Genomic comparisons at the nucleotide level were performed using genome alignment tools MUMmer2 [36], NUCmer [37], and the Artemis Comparison Tool (ACT) [38] (http://www.sanger.ac.uk/Software/ACT/).

PCR and sequencing primers were designed based on draft genome sequences of the A2, F4 and F5 *bont* genes and their botulinum toxin gene clusters (Table S1). PCR was performed with Phusion Hot Start High-Fidelity DNA Polymerase (New England BioLabs, Ipswich, MA) with thermocycling conditions of 98°C for 1 minute and 32 cycles of: 98°C for 20 seconds, 57°C–61°C for 20 seconds and 72°C for 0.5 to 2.5 minutes. Overlapping PCR amplicons were sequenced using an Applied Biosystems Inc, 3730 XL DNA Analyzer (ABI, Foster City, CA). Sanger DNA sequences were assembled with Sequencher software (Gene Codes, Ann Arbor, MI). CLUSTALW multiple alignments of sequences were done with the MEGA5 software [39]. Computation of sequence pairwise identities were done with the EMBOSS pairwise sequence alignment algorithm (http://www.ebi.ac.uk/Tools/psa/).

## Results

### Strain Af84 contains three *bont* gene clusters

Strain Af84, originally isolated from soil from Mendoza Province, Argentina, was sequenced using the Sanger, 454 and Illumina platforms. The sequence was assembled into 43 contigs that totaled 4,086,083 bp. Three of these contigs contained complete botulinum *orfX* toxin gene clusters with *bont* genes for either toxin A2, F4 or F5.

Although the assembled sequences showed the presence of the three *bont*s, the possibility that the original culture might have contained a mixture of *C. botulinum* A2f4 and A2f5 bacteria [3] needed to be examined. Accordingly, total DNA was extracted from 10 single colony isolates of strain Af84. The DNA from each of the 10 colonies was tested by PCR amplification with primer pairs specific for *bont/A* (Primers AF and AR), *bont/F4* (Primers F4F and F4R) and *bont/F5* (Primers F5F and F5R) (Table S1). All 10 PCR amplification reactions confirmed the presence of *bont/A2*, *bont/F4* and *bont/F5* in each of the 10 single colonies of strain Af84 (data not shown).

### Genomic locations of the toxin gene clusters

**Figure 1:**
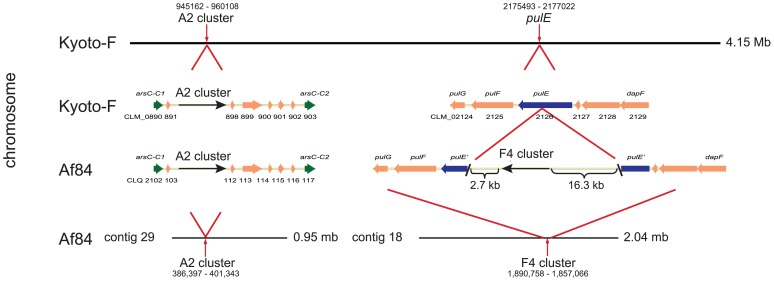
Relative locations of the A2 and F4 toxin gene clusters within the chromosome. The two locations in the chromosome of strain Kyoto-F where the A2 and F4 toxin gene clusters are located within the chromosome of strain Af84 are shown. The A2 toxin gene cluster of strain Af84 is located within two copies of the *arsC* gene (in green), as is the case for the A2 toxin gene cluster of strain Kyoto-F. The F4 toxin gene cluster is located within a split *pulE* gene (in blue). At this site there is no toxin gene cluster within Kyoto-F (or in any other known *C. botulinum* genome). The genomic locations of the toxin gene cluster are magnified with red lines. The horizontal arrows indicate coding sequences (CDSs) and the black arrows indicate the toxin gene clusters. GenBank locus IDs are labeled below the arrows. The first CDS was given the full GenBank locus ID, followed by an abbreviated ID that uses only 2–3 digits.

BLAST search of the contigs of strain Af84 identified high sequence homology to the genome of *C. botulinum* strain Kyoto-F toxin type A2. Figure 1 shows the location of two large contigs in comparison to the Kyoto-F genome (GenBank accession number FJ968749). The Af84 contig 29 (946,263 bp) and the Af84 contig 18 (2,040,448 bp) share 98% and 93% coverage, respectively, and 90–100% sequence identity, with the Kyoto-F chromosome. Each of these contigs, Af84 contigs 29 and 18, contains a *bont* gene.

The *bont/A2* gene cluster within Af84 contig 29 is located within the *ars* operon, between two copies of *arsC*, which is also the location of the *bont*/A2 toxin gene cluster in strain Kyoto-F [18].

The *bont/F4* gene cluster was found in contig 18 (Fig. 1). Alignment of contig 18 with the Kyoto-F chromosome found that between nucleotide positions 2,175493 and 2,177,0022 in the Kyoto-F chromosome, the CLM_2126 gene encodes an intact type II secretion system protein E (*pulE*). Interestingly, in the Af84 contig 18 *pulE* is split at the point where the *bont/F4* gene cluster is located as part of an insert of 34,542 bp (Fig. 1). The two parts of the split *pulE* in strain Af84 can be spliced together with an overlap of 3 nucleotides to create the complete gene.

**Figure 2:**
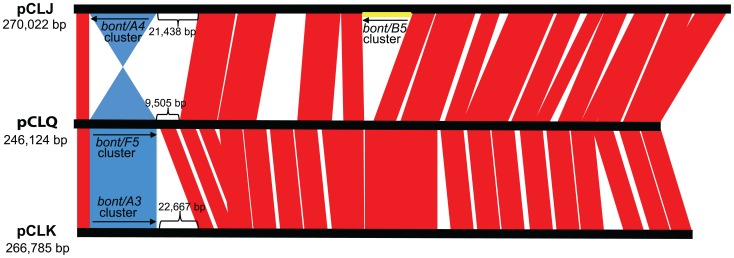
Plasmid synteny among pCLJ, pCLQ and pCLK. The three fully sequenced plasmids are compared by the Artemis Comparison Tool (ACT) [38]. Regions of homology among the pCLJ, pCLQ and pCLK are indicated in red. Regions containing the *bont/A4*, *bont/F5* and *bont/A3 orfX* toxin gene clusters are in blue. The region containing the *bont/B5 ha* toxin gene cluster is in yellow. The comparison shows that the *bont/F5* toxin gene cluster is found in the same location as the *bont/A3* and *bont/A4* (inverted) toxin gene clusters in homologous plasmids.

The *bont/F5* gene cluster of strain Af84 was located within contig 21 (246,124 bp). This contig shows sequence homology with many previously known *bont*-containing plasmids from other *C. botulinum* strains, such as pCLK (*bont/A3* in strain Loch Maree), pCLJ (*bont/A4* and *bont/B5* in strain Ba657) and pCLD (*bont/B1* in strain Okra) (Fig. 2) (GenBank accession numbers CP000963, CP001081 and CP000940, respectively) [18]. A detailed alignment comparison between *bont*-containing plasmids and contig 21 revealed overall synteny and similar location of the *bont/A3*, *bont/A4*, and *bont/F5 orfX* toxin gene clusters (Fig. 2).

We designed PCR primers 1728 bp upstream of the 3′ terminal and 477 bp downstream of the 5′ terminal of contig 21 [F5A3plasmidR2 and F5A3plasmidF1, respectively (Table S1)]. PCR amplification of genomic DNA extract of strain Af84 with these primers produced a product of 2205 bp. The PCR product was purified and sequenced with primers F5A3plasmidF1, F5A3plasmidF2, F5A3plasmidR1 and F5A3plasmidR2 (Table S1). Sequence analysis and alignment of the 2205 bp PCR product and contig 21 found that their terminals were identical and overlapping (5′ to 5′ and 3′ to 3′). Combining the overlapping sequences of the PCR product and contig 21 yielded a closed, circular molecule, thereby confirming that the *bont/F5* gene cluster of strain Af84 was located within a plasmid.

**Figure 3:**
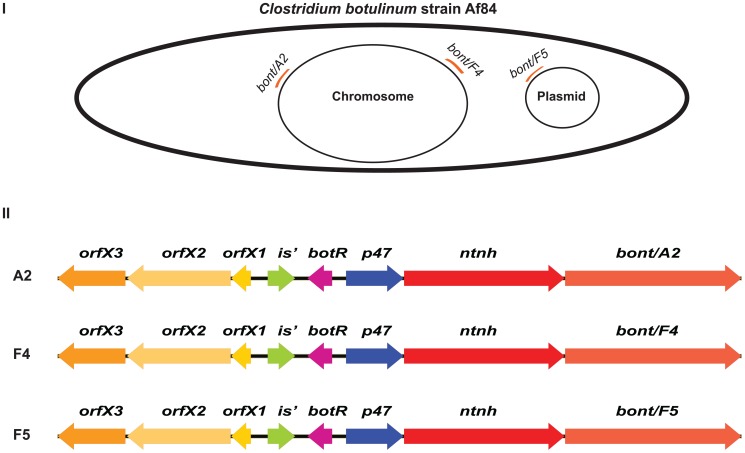
Locations and gene content of the three botulinum toxin gene clusters of *C. botulinum* strain Af84. Schematic presentation of the genomic location of the three botulinum toxin gene clusters in strain Af84 (I) and their gene contents (II). The A2 and F4 toxin gene clusters are located on the chromosome. The F5 toxin gene cluster is located on a large 246,124 bp plasmid. All three toxin gene clusters contain the same genes (*orfX3*, *orfX2*, *orfX1*, *botR*, *p47*, *ntnh* and *bont*) in the same gene arrangement with a 1.2 kb intergenic region, containing a putative degenerated *is* element, between *orfX1* and *botR*.

Figure 3 shows a schematic of a *C. botulinum* strain Af84 cell with the location of the three *bont* clusters (I) and the genetic components of each toxin cluster (II). Each of the toxin clusters contains an *orfX3*, *orfX2*, *orfX1*, a partial insertion sequence (*is*’) element, *botR*, *p47*, *ntnh* and the *bont* gene for either toxin A2, F4 or F5.

### *bont* gene sequence comparisons

The nucleotide sequence of *bont/A2* of strain Af84 (contig 29) was identical to the previously published *bont/A2* sequence (GenBank accession number FJ968749). The nucleotide sequence of the *bont/F5* of strain Af84 (contig 21) was identical to the seven *bont/F5* sequences in GenBank (GenBank accession numbers GU213211, GU213212, GU213215, GU213216, GU213217, GU213222, and GU213225). However, although the nucleotide sequence of *bont/F4* of strain Af84 (contig 18) was identical to the eight other *bont/F4* sequences recently deposited in GenBank (GenBank accession numbers GU213224, GU213223, GU213221, GU213220, GU213219, GU213214, GU213213, and GU213210), it differed by 11 nucleotides from the initial GenBank *bont/F4* accession FJ968748 deposited in 2009.

### Sequence comparisons of the three toxin gene clusters

The three *orfX* toxin gene clusters of strain Af84 had an identical accessory gene content and organization that is recognizable by a 1.2 kb *orfX1*-*botR* intergenic region that contains a putative degenerated *is* element (Fig. 3) [15]. Nonetheless, despite their identical organization, alignment of the regions of the *orfX3* through the *ntnh* (approximately 11 kb) of the three *bont* clusters of strain Af84 identified sequence differences. Specifically, the nucleotide sequence of the F4 toxin gene cluster was only 88.5% and 88.9% identical to the nucleotide sequences of the A2 and F5 toxin gene clusters, respectively. However, the nucleotide sequences of the A2 and F5 toxin gene clusters were more similar with 98.0% identity to each other.

The region of the *orfX3* through the *ntnh* of the A2 toxin gene cluster of strain Af84 was 99.7% identical to the same region of the Kyoto-F strain (GenBank accession number CP001581). The non-identical nucleotides were found almost entirely in the *orfX1*, *orfX2* and *orfX3* genes. The region of the *orfX3* through the *ntnh* of the F4 toxin gene cluster of strain Af84 was most similar (90.2% identity) to the *orfX3*-*ntnh* nucleotide sequence in *C. botulinum* toxin type A3 strain Loch Maree (GenBank accession number CP000963). However, the *orfX2* gene of the F4 toxin gene cluster in strain Af84 had a novel sequence, with less than 68% nucleic acid identity to any *orfX2* of a proteolytic (Group I) strain. Surprisingly, this *orfX2* was most similar to the *orfX2* of nonproteolytic (Group II) *C. botulinum* type E3 strain Alaska 43 (GenBank accession number CP001078), although only with 75.5% nucleic acid identity. We compared the nucleic acid sequence of the other genes of the F4, as well as the A2 and F5, toxin gene clusters to their E3 homologs in strain Alaska and found that they were 66%–85% identical.

**Figure 4:**
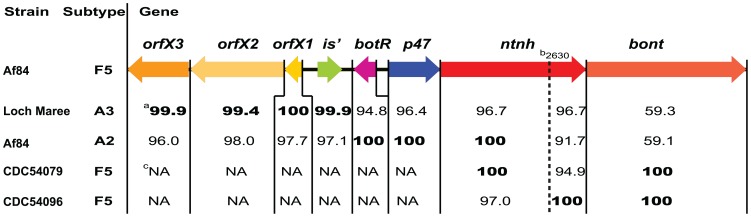
Identification of recombination events in the *bont/F5* toxin gene cluster. Comparison of the nucleic sequences of the *bont/F5* toxin cluster genes of strain Af84 to their homolog genes in other *orfX* toxin gene clusters. The comparison shows that the 5′ terminus of the F5 toxin gene cluster, containing the genes *orfX3*, *orfX2*, *orfX1* and the *orfX1-botR* intergenic region, is almost identical to the 5′ terminus of the A3 toxin gene cluster in strain Loch Maree. In contrast, the 3′ terminus of the F5 toxin gene cluster, containing the genes *botR*, *p47* and most of *ntnh*, is 100% identical to the 3′ terminus of the A2 toxin gene cluster in strain Af84. The *ntnh/F5* and *ntnh/A2* of strain Af84, as well as the *ntnh/F5* of strain CDC5479, are 100% identical until nucleotide position 2630; however, from nucleotide 2631 through the reminder of the gene, the *ntnh/F5* of strain Af84 becomes 100% identical to the *ntnh/F5* of strain CDC54096. The comparisons demonstrate that several recombination events contributed to the creation of the F5 toxin gene cluster in strain Af84. ^a^The numbers below the arrows are percentage identities. Identities higher than 99% are in bold. ^b^nucleotide location within the *ntnh*. ^c^NA, not available in GenBank.

The region of the *orfX3* through the *ntnh* of the F5 toxin gene cluster of strain Af84 was most similar to the *orfX3*-*ntnh* nucleotide sequence of the toxin gene clusters of toxin types A2 and A3. An alignment of each of the genes (and the *orfX1*-*botR* intergenic region) of the F5 toxin gene cluster of strain Af84 with its homolog genes from strains Loch Maree A3 toxin gene cluster, Af84 A2 toxin gene cluster, CDC54079 F5 toxin gene cluster (GenBank accession number GU213215) and CDC54096 F5 toxin gene cluster (GenBank accession number GU213225) is shown in Fig. 4. The 5′ terminus 5,435 nucleotide sequence of strain Af84 F5 toxin gene cluster that contains the genes *orfX3*, *orfX2*, *orfX1* and the 1229 bp *orfX1*-*botR* intergenic sequence is almost identical to the homolog fragment of the A3 toxin gene cluster in strain Loch Maree. However the 4,732 nucleotide sequence of *botR*, *p47* and *ntnh* nucleotide positions 1–2630 of strain Af84 F5 toxin gene cluster is 100% identical to the homolog fragment of the A2 toxin gene cluster in strain Af84 (Fig. 4) and 99.9% identical to the homolog fragment of the A2 toxin gene cluster in strain Kyoto-F. These identities imply that the F5 toxin gene cluster of strain Af84 resulted from a recombination event between the A2 and A3 toxin gene clusters.

The nucleotide sequence of *ntnh/F5* of strain Af84 is 100% identical to the nucleotide sequences of *ntnh/A2* of strain Af84 and to *ntnh/F5* of strain CDC54079 between nucleotide positions 1 to 2630. However, from nucleotide 2631 through the reminder of the gene, the *ntnh/F5* of strain Af84 is only 91.7% and 94.9% identical, respectively, to the *ntnh/A2* of strain Af84 and to the *ntnh/F5* of strain CDC54079 (Fig. 4). Moreover, the nucleotide sequence of *ntnh/F5* of strain Af84 is 100% identical to the nucleotide sequences of the *ntnh/F5* of strain CDC54096 from nucleotide 2602 through the reminder of the gene (891 nucleotides) (Fig. 4). These findings demonstrate that the *ntnh/F5* of strain Af84 resulted from a recombination event.

### Relative amounts of types A and F toxicities

The relative toxicities of the types A and F botulinum neurotoxins in the culture filtrate of strain Af84 were evaluated using the mouse protection assay. Toxicity of culture filtrate diluted 1∶2000 was neutralized by the combination mixture of antitoxins A and F but not by either antitoxin alone. This finding confirmed that strain Af84 produced both (and only) BoNT/A and BoNT/F. The potency of the unabsorbed (no antitoxins) serially diluted culture filtrate was 4.3×106 mouse lethal doses at 50% end-point per ml (MLD50/ml). In contrast, the type F potency of serially diluted culture filtrate mixed with excess CDC monovalent antitoxin A was 6.3×103 MLD50/ml. Accordingly, the ratio of toxins types A:F toxicities in our culture system was 683∶1, and the proportion of culture supernatant toxicity attributable to type F toxin was 0.14%.

## Discussion

The genomic sequencing and analysis of *C. botulinum* strain Af84 identified several unique features: 1) it is the first *C. botulinum* strain that contains three botulinum neurotoxin genes (and three toxin gene clusters, for toxin subtypes A2, F4 and F5); 2) it is the only strain that contains two botulinum neurotoxin genes belonging to the same serotype; 3) it is the first strain that contains more than one *orfX* toxin gene cluster, and 4) it is the first strain that contains botulinum toxin gene clusters within both the chromosome and a plasmid. In addition, the F4 toxin gene cluster of strain Af84 is located at a novel chromosomal site [18].

The three botulinum toxin gene clusters of strain Af84 were located within three separate contigs. The *bont/F5* toxin gene cluster is located within a 246,124 bp plasmid (pCLQ) that is similar to previously known *bont*-containing plasmids from other Group I *C. botulinum* strains (Fig. 2). The *bont/A2* and *bont/F4* gene clusters of strain Af84 were contained within two large contigs (contig 29; 946,263 bp and contig 18; 2,040,448 bp, respectively) that comprise approximately 25% and 53%, respectively, of the chromosome. These contigs shared 98% and 93% coverage, respectively, and 90–100% sequence identity, with the Kyoto-F chromosome. The size and gene content of contigs 29 and 18 led us to conclude that the *bont/A2* and *bont/F4* gene clusters of strain Af84 are located within the chromosome.

In all bivalent *C. botulinum* strains characterized thus far, one of the *bont* genes has been associated with an *ha* toxin gene cluster, while the other *bont* has been associated with an *orfX* toxin gene cluster [18]. Strain Af84 is the first botulinum isolate that contains more than one *orfX* toxin gene cluster. Each of the three *orfX* toxin gene clusters of strain Af84 has an identical genetic content and organization {Fig.3 and [2], [11]}, but the nucleotide sequences of the three *bont* gene clusters of strain Af84 (excluding the toxin genes themselves) were not identical.

Strain Af84 is also the first strain found to contain *bont* toxin gene clusters within both a plasmid and the chromosome. Recently, Hill et al. analyzed the chromosomal and plasmid locations of *bont* toxin gene clusters and found that the clusters were inserted at specific nucleotide locations [18]. In strain Af84 the *bont/A2* toxin gene cluster was located at the same chromosomal site as the *bont/A2* toxin gene cluster in strain Kyoto-F (Fig. 1). This site, an insertion within the *ars* operon, was similar in all *orfX* toxin gene clusters of proteolytic *C. botulinum* strains that have been analyzed. Similarly, the *bont/F5* toxin gene cluster of strain Af84 was located on a plasmid at the previously identified site for other plasmid-borne *orfX* toxin gene clusters (Fig. 2). However, surprisingly, the *bont/F4* toxin gene cluster of strain Af84 was located at a novel chromosomal site within the split *pulE* gene (Fig. 1). The varied, yet non-random, locations of the *bont* gene clusters may reflect insertion and recombination events into the chromosome and plasmids.

The *pulE* codes for a general secretion pathway protein that may be involved in pilus assembly [40]. *pulE* is conserved among proteolytic (Group I) *C. botulinum* strains (>96% nucleotide identity) but is different in other clostridial species with <70% nucleotide identity. Remarkably, we found that the *pulE* of proteolytic (Group I) *C. botulinum* type A3 strain Loch Maree is also split, although at a different position than in strain Af84. The two parts of the split *pulE* in strain Loch Maree type E3 are separated by an insert of 41,733 bp (between chromosome nucleotides positions 2,104,780 and 2,146,513) that does not contain a *bont* gene and that is not homologous to the *bont/F4* insert in strain Af84 or to any sequence in GenBank. The varied content of the *pulE* insert in strains Af84 and Loch Maree demonstrates that these recombination sites are not restricted to *bont*-containing sequences.

We confirmed by the mouse protection assay that strain Af84 produces both BoNT/A and BoNT/F toxins, with almost all (>99%) of the toxicity, under our culture conditions, attributed to the activity of BoNT/A. These results are similar to those of Giménez and Ciccarelli, who found that BoNT/A constituted about 90% to the total toxicity of strain Af84 [26]. Because the factors affecting botulinum toxin production are not completely characterized, different proportions of type A and type F toxicity may be produced in different media and growth conditions. Also, because existing botulinum type F antitoxins neutralize all BoNT/F subtypes, it was not possible to determine either the relative proportions of BoNT/F4 and BoNT/F5 or even that both toxin F subtypes were present in the culture filtrate.

Our characterization of strain Af84 has identified the presence of three toxin gene clusters within a single *Clostridium botulinum* strain. The species may now be considered to be capable of containing either one, two or three botulinum neurotoxin gene clusters. Other *C. botulinum* strains that contain two or more variants of the same toxin type may also exist and be found by combining both cultural and molecular methods.

## Supporting Information

Table S1 **Primers used for amplification and sequencing of the A2, F4 and F5 toxin gene clusters of strain Af84.**
(DOCX)
