# Correction: *Clostridium botulinum* Strain Af84 Contains Three Neurotoxin Gene Clusters: *Bont/A2*, *bont/F4* and *bont/F5*

**DOI:** 10.1371/annotation/b482f80f-c5b6-4b9c-8e9b-8b7139dc37f1

**Published:** 2013-11-04

**Authors:** Nir Dover, Jason R. Barash, Karen K. Hill, Karen W. Davenport, Hazuki Teshima, Gary Xie, Stephen S. Arnon

There are several typographical errors throughout the article. The errors and the correct text are listed below in order of appearance.

In the Title, "*Bont/A2*" should be "*bont/A2*". The full correct title and citation can be found below

*Clostridium botulinum* Strain Af84 Contains Three Neurotoxin Gene Clusters: *bont/A2*, *bont/F4* and *bont/F5*

**Citation:** Dover N, Barash JR, Hill KK, Davenport KW, Teshima H, et al. (2013) *Clostridium botulinum* Strain Af84 Contains Three Neurotoxin Gene Clusters: *bont/A2*, *bont/F4* and *bont/F5*. PLoS ONE 8(4): e61205. doi:10.1371/journal.pone.0061205

In the third paragraph of the Introduction section, in the first sentence, "very rapid recovery" should be "generally very rapid recovery"

In the Materials and Methods section, in the second paragraph of the sub-section "Mouse protection assay", "BoNT/A" should be "BoNT/A2".

In the Materials and Methods section, in the third paragraph of the sub-section "Mouse protection assay", "BoNT/A:BoNT/F" should be "BoNT/A2:BoNT/F".

In the Materials and Methods section, in the third paragraph of the sub-section "Mouse protection assay", "type A" should be "type A2".

In the Results section, in the heading of the sub-section "Relative amounts of types A and F toxicities", "types A and F" should be "types A2 and F".

In the Results section, in the first sentence of the sub-section "Relative amounts of types A and F toxicities", "types A and F" should be "types A2 and F".

In the Results section, in the first paragraph of the sub-section "Relative amounts of types A and F toxicities", "BoNT/A" should be "BoNT/A2".

In the Results section, in the first paragraph of the sub-section "Relative amounts of types A and F toxicities", "4.3x106" should be "4.3x10^6^".

In the Results section, in the first paragraph of the sub-section "Relative amounts of types A and F toxicities", "6.3x103" should be "6.3x10^3^".

In the Results section, in the first paragraph of the sub-section "Relative amounts of types A and F toxicities", "A:F" should be "A2:F".

In the Discussion section, in the fifth paragraph, "type E3" should be "type A3".

In the Discussion section, in the first sentence of the sixth paragraph, both instances of "BoNT/A" should be "BoNT/A2".

In the Discussion section, in the third sentence of the sixth paragraph, "type A" should be "type A2". 

