# Supplementary material for: Clostridium botulinum Strain Af84 Contains Three Neurotoxin Gene Clusters: Bont/A2, bont/F4 and bont/F5
Source: PLoS One. 2013 Apr 12;8(4):e61205. doi: 10.1371/journal.pone.0061205 (PMC3625220; doi:10.1371/journal.pone.0061205)
Supplement: Table S1 — Primers used for amplification and sequencing of the A2, F4 and F5 toxin gene clusters of strain Af84. (DOCX) [file pone.0061205.s001.docx]

**Table S1 - Primers used for amplification and sequencing of the A2, F4 and F5 toxin gene clusters of strain Af84.**

| Primer^1^ | Use^2^ | Sequence | Template(s) | Location^3^ |
| --- | --- | --- | --- | --- |
| A2orfX3F-339 | A | ggaccctacaggcaaaagtg | A2 | 386056-386075 |
| X3R619 | A | TCAAAGTAACCAAGGAGGAGGA | A2, F4, F5 | 386994-387015 |
| X3R231 | A | GGATCATTTTTAGCGGTTGC | A2, F5 | 386608-386627 |
| X3R796 | A | TGACTGATGGCAGAAAAGGA | A2 | 387174-387193 |
| A2X3F-133 | A, S | gagaaggcaagaaattgctca | A2, F4, F5 | 386262-386282 |
| X3R53 | S | GCTTTAGCTGGACCTCTTCAA | A2, F4, F5 | 386430-386450 |
| A2X3R1111 | S | CAAATAATGCAGAAGATCCAGCTA | A2 | 387485-387508 |
| F5X3F406 | S | TCGCAAATAGTTTCACAAATTGA | A2, F5 | 386803-386825 |
| X3F777 | S | TCCTTTTCTGCCATCAGTCA | A2, F4, F5 | 387174-387193 |
| X2R507 | S | ACGTTCAAAAGCAACAACAAT | A2, F5 | 388395-388415 |
| X2R1067 | A, S | CCAAGCCATGCGGTAGATAA | A2, F5 | 388956-388975 |
| A2X2F279 | A, S | ttctttgaccagttgacattgg | A2, F5 | 388186-388207 |
| X2F1406 | A, S | tgttttccgtcattttctcca | A2, F5 | 389313-389333 |
| X2R1665 | S | CCCAAAAGATTTCAAAGCAG | A2, F5 | 389554-389573 |
| F5X2F1112 | S | TGCTTGCATCTAAGTCAGGTTC | A2, F5 | 389020-389041 |
| A2X2R2128 | S | tggaacttcatggaaccaaa | A2, F5 | 390035-390054 |
| X2R1564 | S | caggtgcagatggacgaaat | A2, F5 | 389471-389490 |
| X1R184 | S | AAAAACCGAATGAGTTCACAAA | A2, F4, F5 | 390337-390358 |
| F5X1R402 | S | TGATGACACTTTATCAAATTCTAGTGG | A2, F4, F5 | 390550-390576 |
| X2F1599 | S | CCTGTGGTCATTTCCCAACT | A2 | 389487-389506 |
| X2F1931 | S | TGCCATCTGAATTAGGATTGATT | A2, F4, F5 | 389839-389861 |
| F5X1R-463 | S | TGGAGTGGGAATCTCATACCTT | A2, F4, F5 | 391044-391065 |
| A2orfX1R-673 | A, S | aaatataataccattcatcataatctg | A2 | 391249-391275 |
| F5X1F399 | S | TCCACTAGAATTTGATAAAGTGTCA | A2, F4, F5 | 390549-390573 |
| F5botrR-183 | S | CCCCTGTAGGCGATAAATCA | A2, F4, F5 | 391616-391645 |
| FbotrF448 | S | CTCAAGATAAGCCGTCAATCTG | A2, F4, F5 | 391896-391917 |
| botrF460 | S | CGTCAATCTGTATATAAAAATAAAA | A2, F4, F5 | 391881-391905 |
| FbotRF246 | S | TTGTATGAAAAAGAATAGAGACAAAAA | A2, F5 | 392093-392119 |
| A2botrR363 | S | AAATAAATTTAACACCGAAAATGA | A2, F4, F5 | 392168-392191 |
| F5botrF-261 | A, S | TGCTAGGGAATTTGGCTCTC | A2, F4, F5 | 391392-391411 |
| F5botrF-175 | S | CGCCTACAGGGGTAATTGAA | A2, F4, F5 | 391634-391653 |
| botrR460 | S | TTTTATTTTTATATACAGATTGACG | A2, F4, F5 | 391881-391905 |
| FbotrR448 | S | CAGATTGACGGCTTATCTTGAG | A2, F4, F5 | 391896-391917 |
| A2p47R386 | S | TGCAGGAAGAGGACGAGTTT | A2, F4, F5 | 393049-393068 |
| A2F5p47R495 | S | AAATTGCTTAGGATTCATCCATTC | A2, F4, F5 | 393159-393182 |
| FbotrR246 | S | TTTTTGTCTCTATTCTTTTTCATACAA | A2, F4, F5 | 393093-392119 |
| A2F5p47R807 | S | AGTGCCGCAACTTTAAGTCC | A2, F4, F5 | 393471-393490 |
| F5p47F11 | S | TGGTTGGGATATTGTTTATGGTT | A2, F5 | 392675-392697 |
| A2FntnhR190 | S | AAATTCCTCCATCAGATTTTTGA | A2, F4, F5 | 394097-384119 |
| A2P47F386 | A, S | TGCAGGAAGAGGACGAGTTT | A2, F4, F5 | 393049-393068 |
| A2F5ntnhR304 | A, S | GAAAGGAATAGCCGTGGAAA | A2, F4, F5 | 394234-394253 |
| A2FntnhR308 | A, S | AAGGGAAAGGAATAGCTGTGG | A2, F4, F5 | 394237-394257 |
| A2F5p47F1110 | S | GAAGGGGCATTAGGATTTGA | A2, F4, F5 | 393774-393793 |
| A2FntnhF55 | A, S | gcaattgttagggggagaaa | A2, F4, F5 | 393984-394003 |
| A2FntnhF117 | S | ttgggttgctccagaaagat | A2, F4, F5 | 394046-394065 |
| A2F5ntnhR1285 | S | TCTGCATCATTGATAGGAGGAA | A2, F4, F5 | 395215-395236 |
| A2F5ntnhR1611 | S | CCTCTTGGGTTTCTGTCATATC | A2, F4, F5 | 395541-395562 |
| A2FntnhF767 | S | gtgatgctccgaaaaactttg | A2, F4, F5 | 394696-394716 |
| A2DntnhR2128 | S | TGCTATTTGTGATTCGTTTGAA | A2, F4, F5 | 396029-396050 |
| A2DntnhR2097 | A, S | AGCGGCATTATTAAAAAAACTATCTAC | A2, F4, F5 | 396060-396086 |
| A2FntnhF1154 | S | tttacggggatggcttaaaa | A2, F4, F5 | 395083-395102 |
| A2FntnhF1412 | A, S | aggcccaagtgacaaatagc | A2, F4, F5 | 395341-395360 |
| A2ntnhF5R2635 | S | ggcctaaatttctcaaccaa | A2, F4, F5 | 396545-396564 |
| A2AR90 | A, S | catttgtcctgcatttggaa | A2 | 397523-397542 |
| A2AR201 | A, S | ttgttttgcttctggtggtg | A2 | 397634-397653 |
| A2ntnhF5F2569 | A, S | AATGATTTCTTTGAGAATGGATTA | A2, F4, F5 | 396498-396521 |
| A2AR359 | A, S | tccaccccaaaatggtattc | A2 | 397793-397812 |
| F5ntnhF3024 | A, S | aagggacagtaatgaagaacga | A2, F4, F5 | 396953-396974 |
| A2ntnhF3213 | S | TGTTCAAAAATTTGATGAGGTAA | A2, F4, F5 | 397142-397164 |
| F4R379 | A | CATTAATTGGCGTGTGGTCA | F4 | 1884632-1884651 |
| F4R320 | S | AAAACTTCCCCTGCAGGATT | F4 | 1884573-1884592 |
| F4R54 | S | TGTATCATCATTAACAGGGTCATCA | F4 | 1884302-1884326 |
| F4p47R709 | A | cttgaaagtttaggtaaagctaagt | F4 | 1880189-1880213 |
| F4orfX2F2057 | S | GGTGAGGTCCTTCAGCTTCA | F4 | 1876894-1876913 |
| F4orfX2F1594 | S | CAAGGGCCAAAAGTACCATC | F4 | 1876431-1876450 |
| F4orfX2R2227 | S | CAAATCGCATAAGGGAGGAA | F4 | 1877095-1877114 |
| F4orfX2F1042 | S | ACCGCATGATTTGGTCTAGC | F4 | 1875879-1875898 |
| F4orfX2R2070 | S | TGAAGGACCTCACCCTGTTC | F4 | 1876888-1876907 |
| F4orfX2R1879 | S | TTTTAAATGTTTCGGAAGACGA | F4 | 1876698-1876719 |
| F4orfX2R1767 | S | TCAAACTAGAGTTGAAGGGGATT | F4 | 1876582-1876604 |
| F4orfX2F418 | S | TCTGTACCCATGTTATCCCAAA | F4 | 1875255-1875276 |
| F4orfX2R1364 | S | AACGCTGTTATAATCGTTGGTTC | F4 | 1876179-1876201 |
| F4orfX3F1466 | S | TCGTTTGCATTAACCTTGACA | F4 | 1874801-1874821 |
| F4orfX3F1300 | S | CCTCCATCTCCCCCACTTAT | F4 | 1874635-1874654 |
| F4orfX2R775 | S | AAATAACCGACGCAACTTGG | F4 | 1875593-1875612 |
| F4orfX3F992 | S | CCATGACAAATTCATCATCTTCA | F4 | 1874327-1874349 |
| F4orfX2R231 | S | GCAGCTGGTATAGCATCATCA | F4 | 1875068-1875088 |
| F4orfX3F597 | S | TCCTCCTCCTTGGTTACTCTGA | F4 | 1873932-1873953 |
| F4orfX2R190 | S | TGTCAAGGTTAATGCAAACGA | F4 | 1874801-1874821 |
| X3F25 | A, S | tcctcctaattgaagaggtcca | F4, F5 | 1873359-1873380 |
| A3X3F-81 | S | ggaggaaattaaaaagcataagg | F4 | 1873232-1873254 |
| A4orfX3-659 | A, S | tgccttgaaggtatgtgcag | F4 | 1872974-1872993 |
| F4p47X3F-427 | S | gggggtttttaggaatggaa | F5 | 14077-14096 |
| A3orfX3F-249 | A, S | tggcttgaaggtgtaaaattatg | F5 | 13963-13985 |
| F5orfX3F-427 | S | gggggtttttaggaatggaa | F5 | 14077-14096 |
| orfX3R1230 | S | TACAGGGGGTTTCGAATCTG | F5 | 15716-15735 |
| ntnhF5X2R700 | S | CAAAATCTGGAAAGCAAATTCC | F5 | 16695-16716 |
| F5_X2F1559 | S | CCTGTGGTCATTTCCCAACT | F5 | 17595-17614 |
| F5orfX1-421 | A, S | TTTGACATATATAGTATTGGCAGTT | F5 | 19107-19131 |
| P47R190 | S | tccttccttaattggcgtctt | F5 | 20965-20985 |
| bontR820 | S | CATCGTTCCCTCCAAAAGTTAG | F5 | 26340-26361 |
| ntnhF5R3336 | S | aatgagcattttctttgcact | F5 | 25357-25377 |
| ntnhF5R | S | TTTCAACTGGCATAAAAATCC | F4, F5 | 1884265-1884285 |
| bontF5R215 | A, S | tatccatcagaccctgcttt | F5 | 25740-25759 |
| bontF5F152 | S | ggcttggtatagaggcttca | F5 | 25696-25715 |
| AF | A | aggttaaTacaCagattgatctaataag | A2 | 399611-399638 |
| AR | A | tagcaTcaaaatcttytaaccgtttaac | A2 | 399865-399892 |
| F4F | A | GCTTTAAATATAGGTAATGATGCAAG | F4 | 1886154-1886179 |
| F4R | A | TAATGATTTTATCTTCATTCTTAGATG | F4 | 1886296-1886322 |
| F5F | A | GCCAGGACCCGATTTAACTAC | F5 | 26015-26035 |
| F5R | A | CATCGTTCCCTCCAAAAGTTAG | F5 | 26340-26361 |
| F5A3plasmidF1 | A, S | caccaattggatgactatcatgt | F5 | 245648-245670 |
| F5A3plasmidF2 | S | tgcttcttcctcattgtttgc | F5 | 245895-245915 |
| F5A3plasmidR1 | S | aaaagtattcagcgggaagaaa | F5 | 1069-1090 |
| F5A3plasmidR2 | A, S | taacagcaataagagagaagggat | F5 | 1705-1728 |

^1^The last letter in primer name indicates direction (F=Forward, R=Reverse).

^2^Primers usage: A = primer used for amplification; S = primer used for sequencing;

^3^The location of the primer sequence according to the contig containing the first toxin type in the Template column.
